# Detection of Safflower Adulteration in Saffron Using Ion Mobility Spectroscopy

**DOI:** 10.1155/jamc/6366923

**Published:** 2025-05-27

**Authors:** Mahtab Heyrani, Mohammad-Taghi Golmakani, Mohammadreza Khalesi

**Affiliations:** ^1^Department of Food Science and Technology, Shiraz University, Shiraz, Iran; ^2^Department of Biological Sciences, University of Limerick, Castletroy, Limerick V94 T9PX, Ireland

**Keywords:** eugenol, ion mobility spectrometry (IMS), safflower, saffron adulteration

## Abstract

Saffron (*Crocus sativus* L.), an exceptionally valuable and expensive spice on an international scale, has become the target of a rapid increase in fraudulent practices. In an effort to decrease expenses, stigmas of safflower (*Carthamus tinctorius*), which closely resemble saffron, are often added to pure saffron as a typical method of adulteration. Hence, by quantifying the extent of eugenol modifications in the samples and employing ion mobility spectrometry (IMS) to identify and quantify these adulterants in saffron, the objective of this research has been accomplished. The analysis of eugenol showed a significant increase in peak intensity as the concentration of safflower increased in laboratory-prepared samples of pure saffron and safflower as well as the mixture of them (25%, 50%, 75%, and 100%, v/v). In the subsequent phase, a total of 20 saffron samples procured from nearby markets were examined under an optical microscope to identify any adulteration with safflower. Five samples, which included saffron containing safflower at varying concentrations (8.3%, 14.9%, 19.4%, 25.4%, and 33.7% W/W), were chosen for additional IMS analysis. The results showed that the peak intensity of eugenol climbed from 0.20 to 0.28 mV by augmenting the safflower content in saffron. Therefore, by increasing the level of safflower contamination in saffron, the concentration of eugenol in the IMS rose. The outcomes demonstrated that the selection method effectively detects saffron adulterated with safflower, improving both precision and specificity, and could aid in defining standard quality control procedures for saffron authenticity and quality.

## 1. Introduction

Saffron is a perennial plant that usually grows in semi-arid and dry areas. It is popular for its fragrant smell, taste, color, and unique medical features and is considered an important food additive. It has a significant economic impact on Iran's agricultural exports; in 2020, it was the world's largest saffron exporter, bringing in $108 million [1]. Saffron ranked 18th among Iran's most exported products in 2020, and the main destinations of saffron exports from Iran were Hong Kong ($33.6 million), Spain ($26.9 million), China ($11.1 million), the United Arab Emirates ($8.81 million), and Kuwait ($8.72 million) [2]. An estimated 300 tons of saffron are produced annually on a global scale, with the majority of production countries being Iran (which provides about 88% of the global supply), India, Spain, and Greece [3]. Saffron has consistently been vulnerable to fraudulent activities motivated by economic considerations as a result of its long history of adulteration, totaling over six centuries [4]. Adulterating saffron can be achieved through a range of techniques. These include combining it with older or condensed saffron, blending it with other floral species such as safflower, calendula, and marigold, adding styles and stamens, incorporating heavier substances (e.g., syrup, glycerin, olive oil, and honey), and supplementing powdered saffron with substances such as turmeric, paprika, salts, and synthetic dyes that resemble familiar substances [5–8].

Safflower, scientifically known as *Carthamus tinctorius*, is frequently used as an adulterant in place of saffron due to the visual similarity and unique aromatic properties of the two substances [9]. Constituting an essential commercial crop, this plant is classified as a member of the Asteraceae or Compositae family. It finds application as a dye source, oilseed livestock feed, biofuel production, and medicinal purposes, such as the improvement of cerebral blood flow and the treatment of a range of ailments including cardiovascular, gynecological, and cerebrovascular disorders, hypertension, and coronary heart disease [10]. Safflower was most likely domesticated more than 4000 years ago in the fertile Mediterranean coastal zone, and it was grown before the widespread availability of less expensive aniline dyes for use as textile staining dyes [11]. Eugenol is a prevalent compound present in safflower. This compound is a propanoid that has a strong smell and a molecular weight of 164.2 g.mol^−1^, which may dissolve in organic solvents. Eugenol is mostly derived from clove oil (70%–90%), as well as from sugar beet, cinnamon, basil, and bay leaves [12–15]. It has a pleasant, spicy, and clove-like aroma and is used in the pharmaceutical, food, and cosmetic industries in limited concentration. Eugenol derivatives are used in medicine as an antibiotic and local anesthetic [16–18]. Traditional, biochemical, and microscopic approaches may be used to assess the purity and quality of saffron. However, these procedures have some drawbacks, including limited accuracy and sluggish reaction times. Therefore, it is imperative to investigate precise and efficient techniques for assessing the purity of saffron.

**Figure 1:**
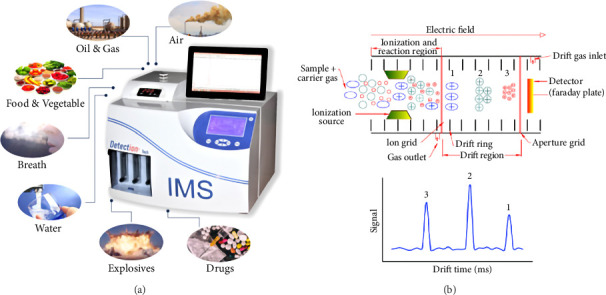
(a) The IMS device image utilized in this research and its various applications; and (b) the schematic.

Today, various methods, including thin layer chromatography, liquid chromatography/mass spectrometry, near-infrared spectroscopy (NIR), image processing techniques, Gas Chromatography/Mass Spectrometry (GC-MS), electronic nose, and nuclear magnetic resonance, are used to identify impurities in saffron [19]. NIR spectroscopy is applied for the detection and quantification of eight different adulterants found in saffron (50 samples) collected from Egypt. These adulterants include calendula, curcuma, exhausted saffron, hibiscus, pomegranate peel, paprika, safflower, and stamens of saffron. The NIR spectra of saffron samples and adulterated saffron, which was created by combining individual adulterants in amounts varying from 10 to 400 mg.g^−1^, were obtained within the 12,000–3500 cm^−1^ range. Detecting low (1%) levels of adulterants was the most effective with the wavenumbers of 4600–4200 cm^−1^, 5400–5000 cm^−1^, and 6000–5800 cm^−1^ [20]. GC-MS was used to identify authenticity, find adulterants, and evaluate how roasting affected the aroma of saffron flowers Farag et al. [21]. A significant difference between saffron and its less-graded spices was also observed, as was the impact of roasting on saffron. In order to distinguish saffron from its two allied flowers, safranal and 2-caren-10-al were found to be discriminatory markers [21]. There are several techniques that can be used in order to recognize saffron fraud, but most of them are costly, specific to adulterating compounds, time-consuming, and require a high level of technical expertise. This has led to the development of rapid detection methods for real-time quality control, especially for fast detection of adulteration in processed foods, which are cheap and efficient. IMS is an instrumental analysis method that is somewhat similar to time-of-flight (TOF) mass spectrometry, except that it operates at atmospheric pressure [22, 23]. It is utilized in an unprecedented number of fields, including the rapid analysis of food ingredients, on account of its rapid scanning time and capability to distinguish between positive and negative ions (Figure 1). Moreover, in order to aid in the identification of unidentified compounds, this practical instrument can provide precise masses as well as potential elemental compositions of precursors and fragments [24].

The IMS approach was employed in a study to identify the presence of impurities in canola oil samples. The examination encompassed 147 samples of canola oil and focused on detecting adulteration in other vegetable oils such as sunflower, soybean, and peanut oils. Partial least squares (PLS) analysis was employed to forecast the quantities of adulterant oil in canola oil. The study revealed that the PLS model proved to be an effective method for predicting adulteration, with strong regression (*R*^2^ > 0.95) and minimal errors (root-mean-square error [RMSE] ≤ 3.23) [25]. During a separate investigation, IMS was employed to detect adulteration in sesame oil. The IMS device was used to evaluate sesame oil samples that were injected with diluted hexane solvent. Subsequently, chemical techniques were employed to determine the suitable model for distinguishing between pure sesame oil and adulterated sesame oil mixed with four other edible oils. The results demonstrated that the Support Vector Machine using R (R-SVM) model was highly effective in detecting sesame oil samples containing above 10% fraudulent content, with an accuracy rate of 94.2% [26]. In another study, in order to classify olive and camellia oils quickly and easily, IMS fingerprints were used along with a chemometric model (peak detection and a random forest algorithm). Based on a random forest algorithm, a discriminant model was established to detect olive oil adulterated by camellia oil. Simulation showed that 96.4% of 55 samples were identified as blending olive oil with camellia oil [27].

In recent years, there has been a significant surge in the number of papers on IMS applications in routine analysis for detecting and preventing fraud. Five specific bio-adulterants derived from plant sources—safflower, calendula, curcumin, sumac, and saffron stamens—were utilized in a research investigation to determine whether or not saffron had been adulterated. In varying proportions (0%–50%), the bio-adulterants were introduced into 10 distinct saffron preparations. For the prediction of adulteration ratios of saffron, calendula, curcumin, safflower, and sumac stamens, PLS regression yielded promising findings. The correlation between multiple determinations (*R*^2^) and RMSE for the test set samples varied from 4.82 to 10.33% [28]. An additional inquiry employed multivariate data analysis and spectral fingerprints produced by an IMS to identify instances of saffron adulteration involving synthetic edible colorants, including tartrazine, sunset yellow, Ponceau 4-R, and erythrosine, using user-friendly procedures. Due to this rationale, synthetic colorants were introduced into 10 samples of saffron in different proportions ranging from 0% to 30% (w/w). Principal component analysis (PCA) was employed to account for 92.28% of the data variance, enabling a reasonable level of differentiation to be established between the saffron samples and the remaining four synthetic dietary colorants. Furthermore, the RMSE and correlation of the multiple determination (*R*^2^) values for the test set mixtures consisting of saffron and synthetic edible colorants were found to be 2.39%–3.53% and 0.880–0.954, respectively [29].

IMS has not been used to detect safflower adulteration in saffron, to the best of our knowledge. The aim of this study was to measure the eugenol content with an IMS device in order to determine the amounts of safflower adulteration in different saffron samples. To begin with, the amount of eugenol in each of the samples prepared in the laboratory, namely pure saffron, safflower, and mixtures of saffron and safflower, was calculated. In the next step, the amounts of eugenol in saffron samples purchased from the market (containing different concentrations of safflower) were determined.

## 2. Materials and Methods

### 2.1. Materials

Premium stringy saffron was purchased from Novin Saffron Company (Mashhad, Iran). Safflower had been bought from a market specializing in herbal medicines in Shiraz, Iran. A quantity of hexane, ethanol, diethyl ether, and pure eugenol (99.9%) were acquired from Merck, a German company.

### 2.2. Choosing Suitable Solvent for Extraction

In order to get the extracts of safflower and saffron, a variety of solvents were utilized. These solvents included methanol, ethanol, diethyl ether, hexane, and water. At this point in the process, the peak of the solvent should not coincide with the peak of the eugenol substance.

### 2.3. Analytical Parameter Evaluation

The application was carried out using an IMS device that was equipped with a corona discharge source (IMS-300, TOF Tech.Pars Co., Ltd.; Isfahan, Iran). In order to achieve the optimal eugenol peak, the IMS is optimized by taking into account various variables. These variables include the speed of the carrier gas flow (300 mL.min^−1^) and the drift gas (700 mL.min^−1^), the voltage applied for ionization (6000, 7000, and 8000 V) and the drift region (437.5 V cm^−1^), the temperature of the injection region (200, 230, and 260°C), and the cell temperature (140, 170, and 200°C). The procedure for injecting the sample consisted of the following steps: First, a mixture of 10 mL of solvent and 1 μL of pure eugenol was combined and stirred for a duration of 2–3 min using a vortex. After the preparation of the solution, a volume of 10 μL was injected into the IMS device. Following the drying of the solution via thermal means, the corresponding peak was identified. In order to preserve the data, the software known as PicoScope 2204 (which was developed by Pico Technology in the United Kingdom) was installed. After recording the data for a period of 5 minutes, the mean curve was then gathered.

### 2.4. Eugenol Quality Test

To ascertain the retention period of eugenol ions in the spectrum, solutions with differing concentrations of pure eugenol (i.e., 100, 500, 1000, 2000, and 2500 ppb) dissolved in hexane were injected. To thoroughly dissolve the eugenol, the tube holding the eugenol solution was put in a shaking incubator at a speed of 2000 rpm for a duration of 15–20 min. Subsequently, the solutions were injected using the most favorable parameters of cell temperature, injection temperature, and voltage. Therefore, the retention time of the peak related to eugenol was identified. Thus, the calibration curve for eugenol was obtained.

### 2.5. Preparation and Extraction of Reagents Sample

With the aim of measuring the amount of eugenol, its extract must be extracted from saffron and safflower. In order to fulfill the objective, the laboratory has prepared samples of pure saffron, pure safflower, and a combination of the two in varying ratios of 25:75, 50:50, and 75:25. To obtain the extract from the samples, a total of five solvents were taken into consideration, namely hexane, ethanol, methanol, diethyl ether, and water. A mixture of 25 mg of the sample and 10 mL of the solvent was agitated at 37°C and 2000 rpm for a duration of 5 hours utilizing a magnetic stirrer in order to ascertain the most suitable solvent. Then, the solution was filtered using filter paper (0.45 Mm) and stored in the refrigerator for further analysis [30]. Safflower and saffron extracts (with ratios of 0:100, 25:75, 50:50, 75:25, and 100:0) were injected into the IMS device. The spectral characteristics of sample extracts were monitored by scanning from 0 to 10 ms using the IMS controlled by the PicoScope. Through a comparative analysis of the eugenol spectrum and other solvent spectra, the most suitable solvent was identified. Based on the intensity of the obtained peaks and by comparing them with the standard curve of eugenol, the amounts of eugenol extracted from different concentrations were measured.

### 2.6. Detection of Saffron Adulteration in Market Samples

Twenty saffron samples bought from a nearby market were subjected to a 10x magnification examination under a microscope in order to ascertain the percentages of safflower adulteration present in the samples. A total of 20 distinct locations on the saffron samples were selected at random for photography and subsequently subjected to analysis using Image J software (1.52v, Bethesda, Maryland, USA). IMS was then used to determine the eugenol content of the five samples selected from the 20 samples. Furthermore, an analysis was conducted to compare the outcomes acquired using an optical microscope, which is the conventional approach.

### 2.7. Statistical Analysis

The software application utilized to compute the mean (*n* = 3) and standard deviation (SD) was Microsoft Office Excel 2016. Furthermore, Statistical Analysis System (SAS) software was employed to carry out experiment design and statistical data analysis.

## 3. Results and Discussion

### 3.1. Solvent Selection

**Figure 2:**
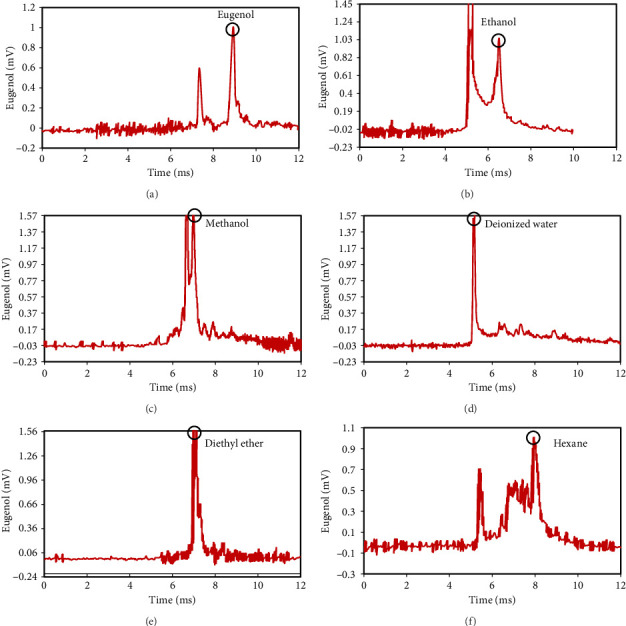
Ion mobility spectrometer spectra of eugenol (a), ethanol (b), methanol (c), deionized water (d), diethyl ether (e), and hexane (f).

The peak of pure eugenol was observed at 8.5 min (Figure 2(a)); thus, a suitable extraction solvent is the one without an overlap peak at this time as eugenol (i.e., without any interference). Except hexane, all solvents (ethanol, pure methanol, deionized water, and diethyl ether) showed peaks at this time, indicating the contamination of the device or overlap with eugenol (Figures 2(a), 2(b), 2(c), 2(d), 2(e), 2(f)). As a result, they were unsuitable for extraction. The hexane peaks had no spectral overlap with the eugenol peak, which was situated beyond the contaminated region of the device. Furthermore, hexane effectively extracts optimal quantities of eugenol from safflower and saffron (Table 1). Thus, hexane was selected as a proper solvent for the extraction and measurement of eugenol using IMS. Farag et al. [21] used different solvents (methanol, acetone, ethanol, and hexane) to prepare saffron extract for online monitoring of solvents by IMS. The results showed that hexane was the best solvent since it extracted eugenol at the highest content.

### 3.2. Optimization of IMS Conditions

In order to achieve the most accurate results while measuring eugenol using IMS, a number of adjustments were made to the voltages, cell temperatures, injection temperatures, and flow rates of carrier, buoyancy, and dopant gases. The ideal values and optimization indices were as follows: 8 kV, 200°C, 260°C, 300 mL.min^−1^, 600 mL.min^−1^, and unusable, respectively.

#### 3.2.1. Cell Temperature

**Figure 3:**
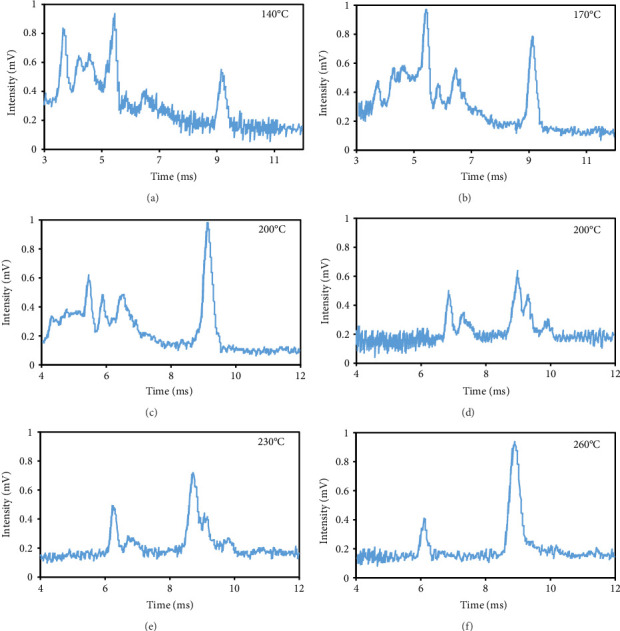
The impact of cell temperatures on eugenol peak intensity (a–c) and different injection temperatures affects eugenol peak intensity (d–f).

The peak duration and intensity are both affected by the temperature of the cell (Figures 3(a), 3(b), 3(c)). The retention period was reduced as a result of a rise in the temperature of the cell, but the intensity of the eugenol peaks was enhanced. The decrease in peak duration can be attributed to the correlation between temperature and molecular density within the cell. An increase in temperature promotes higher mobility, which in turn decreases the time required for ions to reach the detector. Consequently, this results in shorter drift times, which are indicative of peaks appearing more frequently. The shorter time required for the ions to reach the detector, resulting in a decrease in their loss rate and an increase in their passage rate, may also contribute to the increase in peak intensity.

#### 3.2.2. Injection Temperature

The optimum temperature for injection into the IMS is different for each substance and depends on its stability and melting point. The magnitude of the observed peak intensified as the injection temperature was raised (Figures 3(d), 3(e), 3(f)). The temperature of 260°C exhibited the greatest peak intensity, potentially due to the increased rate of eugenol decomposition at elevated temperatures. Eugenol's decomposition commenced at a temperature of 254°C, which is its melting point. As a result of the accelerated decomposition and increased number of components produced at elevated temperatures, eugenol exhibited an increase in the intensity of the peaks associated with the decomposed components. The optimal injection temperature was determined to be 260°C, which was the maximum applicable temperature of the injection area in the IMS device.

#### 3.2.3. Applied Voltage

Utilizing two high-voltage Direct Current (DC) power sources, an electric field and corona were generated in the drift region. It is possible to enhance the initial power supply by as much as 8 kV via the device panel. The constant voltage of the second power supply, which was isolated, was 2300 V. The potential difference along the entire length of the cell was generated by the first power supply, whereas the potential difference in the corona electrode was supplied by the second power supply. The residence time of the peaks was prolonged and the field was diminished through the reduction of the drift voltage. The signal, however, became weaker due to a reduction in the quantity of ions that reached the end of the cell. At a voltage of 8 kV, the electric field strength and the number of ions that reached the end of the cell increased, indicating that 8 kV is the optimal voltage for this project.

#### 3.2.4. Effect of Dopant Gas

The application of ammonia dopant gas as an enhancer was investigated to increase the intensity of the eugenol peak. Ammonia vapor was injected into the ionization section, so the peaks of H_3_O^+^ and NO^+^ were completely removed, and only the peak of NH_4_^+^ was recorded. Finally, the results indicated that the use of ammonia dopant gas had no significant effect on increasing the intensity of the eugenol peak.

### 3.3. Eugenol Quality Test

**Figure 4:**
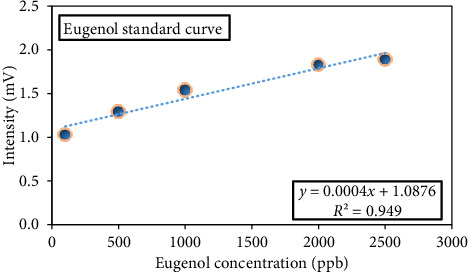
Eugenol standard curve.

The duration of the eugenol peak was determined for various concentrations. Once the exact position of the peak was confirmed, eugenol was chosen as an indicator, and a standard curve was created. The pure eugenol solution was prepared using hexane solvent at different concentrations of 100, 500, 1000, 2000, and 2500 ppb. Then, the solutions were injected into the IMS under optimum conditions of cell temperature (200°C), injection temperature (260°C), and voltage (8 kV). As the concentration of eugenol was increased, there was a corresponding increase in the intensity of its peak (peak intensity = 0.0004 eugenol concentration + 1.0876, *R*^2^ = 0.949) (Figure 4) [31].

### 3.4. Adulteration Detection Using the Eugenol Index

**Figure 5:**
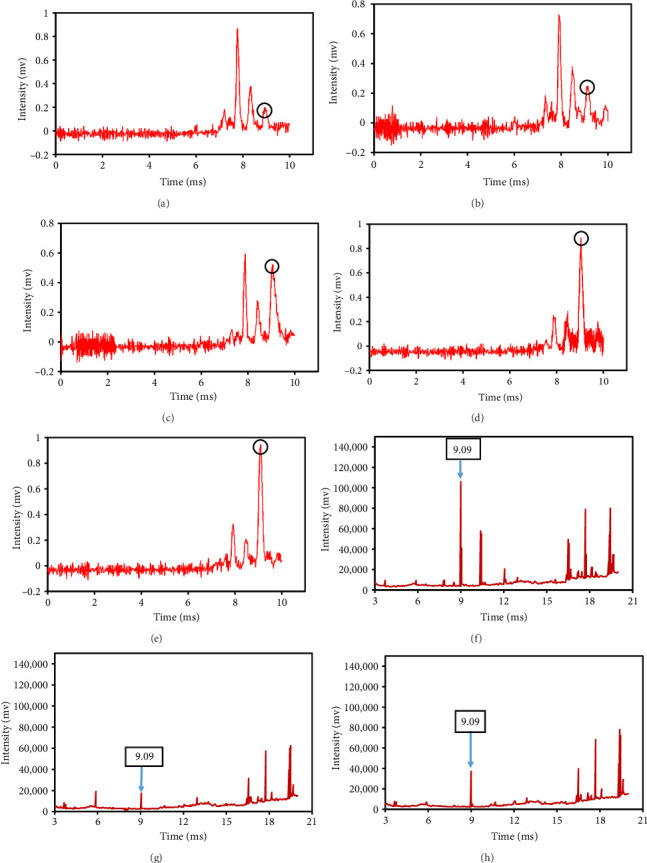
Eugenol spectrum of (a) saffron sample as well as saffron samples with (b) 25%, (c) 50%, (d) 75%, and (e) 100% safflower. Chromatograms of saffron containing different concentrations of safflower: (f) 0%, (g) 75%, and (h) 50%.

The quantification of extracted eugenol was determined by comparing the peak intensities of various quantities of eugenol injected into the IMS with the eugenol standard curve. This demonstrated that the amount of safflower in the saffron mixture was the underlying factor. Figures 5(a), 5(b), 5(c), 5(d), 5(e) display the spectra of eugenol obtained from safflower and saffron samples with varying ratios. Based on the acquired chromatograms, a direct relationship was seen between the strength of the eugenol peaks in various samples and the safflower content in those samples. Different intensities of peaks were obtained by injecting saffron samples that were adulterated with varying quantities of safflower into the IMS. The intensity of the eugenol peak varied at 0% (Figure 5(a)), 25% (Figure 5(b)), 50% (Figure 5(c)), 75% (Figure 5(d)), and 100% safflower (Figure 5(e)), respectively, with values of 0.175, 0.228, 0.505, 0.886, and 0.995 mV. An increase in the amount of safflower adulteration in saffron resulted in a correspondingly significant augmentation in the quantity of eugenol [32]. Although the concentrations of eugenol in safflower samples vary, it is consistently observed that the eugenol levels in various safflower varieties are considerably higher than those in saffron varieties. As a result of its exceptional sensitivity, the IMS device precisely determines the eugenol concentration. IMS was utilized to quantify heavy metals in potable water, which is consistent with our findings. The intensity of the corresponding peaks in potable water increased as the concentration of heavy metals was elevated [33]. Further, IMS analysis detected explosives in the water samples, even at the ppt level [23].

This study evaluated chromatograms of saffron samples with differing amounts of safflower. These chromatograms distinctly demonstrate the alterations in the chemical profiles of the samples according to the proportion of safflower admixture.  Figure 5(f) depicts the chromatogram of pure saffron (0% safflower), Figure 5(g) exhibits saffron combined with 75% safflower, and Figure 5(h) represents the chromatogram of saffron blended with 50% safflower. The chromatograms clearly demonstrate variations in the chemical composition of the samples, offering a dependable technique for identifying and quantifying the extent of safflower adulteration in saffron. The findings from this research can be employed for quality control and evaluating the purity of saffron. This method is very beneficial for verifying the authenticity of saffron in both commercial and scientific contexts.

### 3.5. Eugenol Measurement of Market Samples

**Figure 6:**
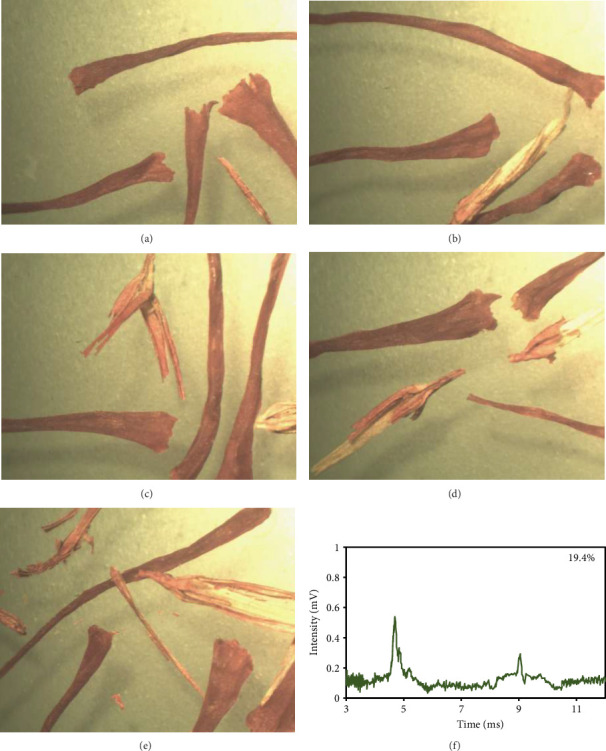
Images of saffron market samples taken under optical microscope. Saffron samples with safflower adulteration levels of (a) 8.3%, (b) 14.9%, (c) 19.4%, (d) 25.4%, (e) 33.7% and spectrum of saffron sample as well as saffron samples with 19% of safflower (f) are shown.

At first, a total of 20 saffron samples were randomly acquired from the market and examined using an optical microscope. After analyzing the photos of the saffron samples, it was determined that 5 of the samples were adulterated, while the remaining 15 samples were not contaminated with safflower. The optical microscope analysis revealed that the saffron samples included safflower in the proportions of 8.3%, 14.9%, 19.4%, 25.4%, and 33.7%.  Figure 6 displays images depicting the varying quantities of safflower present in saffron samples. The safflower content of the samples was analyzed using the IMS technique by examination with an optical microscope in the subsequent stage.  Table 2 shows that the response intensity values were 0.2067, 0.2400, 0.2533, 0.2667, and 0.2867 for samples containing 8.3%, 14.9%, 19.4%, 25.4%, and 33.7% safflower, respectively. The results showed that the strength of the IMS response correlated positively with the safflower content in the saffron samples.  Figure 6(f) displays the spectrum derived from IMS of the commercial saffron sample containing 19.4% safflower, in which the eugenol spectrum is distinctly visible, akin to that of the laboratory samples.

**Figure 7:**
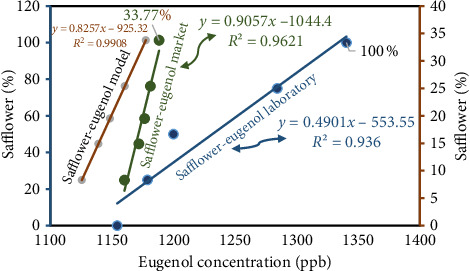
Safflower and eugenol concentrations in lab, market, and model samples.

Ultimately, the IMS device was employed to examine the saffron samples obtained from the market as well as the samples prepared in the laboratory in order to ascertain the quantity of eugenol present in each sample. Initially, five saffron samples containing different concentrations of safflower (0%, 25%, 50%, 75%, and 100%) were injected into the IMS device. Subsequently, market samples with safflower concentrations of 8.3%, 14.9%, 19.4%, 25.4%, and 33.7% were also injected. The determination of eugenol concentrations in various saffron samples can be accomplished by substituting the corresponding eugenol peak intensities into the standard curve equation. The eugenol concentration in market saffron samples increased from 1160.772 to 1188.260 ppb as safflower levels rose from 8.3% to 33.7% (Table 3). The equation utilized as the foundational model in this investigation is obtained from laboratory samples. The eugenol concentration in the samples is ascertained and documented in Table 3 using the aforementioned equation.  Figure 7 illustrates that an increase in safflower concentration results in this consequence in both market and model samples, as determined by a comparative analysis of the eugenol concentrations in those samples.

## 4. Conclusion

The objective of this study was to quantify the concentration of eugenol using IMS as a means of identifying safflower adulteration in saffron samples. To begin working toward this objective, the initial task was creating saffron samples with varying percentages of safflower (ranging from 0% to 100%) in the laboratory. Subsequently, the IMS system was fine-tuned to accurately quantify the levels of eugenol in these samples. Five adulterated safflower samples were subsequently chosen after an optical microscope examination of several market saffron samples. Using Image J software and an optical microscope, the concentrations of safflower in market samples were ascertained in the next step. The concentration of eugenol in market samples was determined using IMS analysis. Saffron market samples containing 8.3%, 14.9%, 19.4%, 25.4%, and 33.7% percent safflower had eugenol concentrations of 1160.772, 1171.864, 1176.533, 1181.238, and 1188.260 ppb, respectively. The findings verified that raising the safflower concentration resulted in an increase in the peak intensity of eugenol. The conclusion drawn from the data from the IMS analysis and optical microscopy methods is that elevating the safflower level raised the concentration of eugenol, which may have contributed to the adulteration of saffron. Therefore, the high-speed performance of the IMS device is essential for this study, and its exceptional sensitivity at the ppb level enables amateurs to prepare samples in a wide range of situations. This method has great potential for detecting food and medication adulteration due to its ability to utilize various ionization sources and be combined with GC, GC-MS, and HPLC.

## Figures and Tables

**Table 1:** Extraction percentage of eugenol with different solvents.

Solvent	Extraction yield (%)
Diethyl ether	4.1
Deionized water	6.3
Methanol	7.8
Ethanol	8.9
Hexane	9.7

**Table 2:** Response of the ion mobility spectrometry (IMS) to the saffron adulteration with safflower.

Safflower concentration (%, wt/wt)	IMS response
8.3	0.2067 ± 0.04^∗^
14.9	0.2400 ± 0.03
19.4	0.2533 ± 0.04
25.4	0.2667 ± 0.05
33.7	0.2867 ± 0.02

^∗^Mean ± SD (*n* = 3).

**Table 3:** Eugenol concentration obtained from saffron samples adulterated with safflower by experiment and prediction model.

Saffron (%)	Safflower (%)	Eugenol concentration (ppb)		Error (%)
		Market	Model	
91.7	8.3	1160.772	1129.885	−2.61
85.1	14.9	1171.864	1129.953	−3.58
80.5	19.5	1176.533	1129.980	−3.96
74.6	25.4	1181.238	1130.007	−4.34
66.3	33.7	1188.260	1130.048	−4.90

## Data Availability

The data that support the findings of this study are available from the corresponding author upon reasonable request.
